# Biguanides and targeted anti-cancer treatments

**DOI:** 10.18632/genesandcancer.59

**Published:** 2015-03

**Authors:** Elyse K. Hanly, Zbigniew Darzynkiewicz, Raj K. Tiwari

**Affiliations:** Department of Microbiology and Immunology, New York Medical College, Valhalla, NY

Drug repositioning and molecularly targeted novel drug development are two contrasting strategies in cancer research. Yet, certain combinations of the two may bring together the best of both worlds. Drug repositioning, i.e. the use of a drug for treating diseases other than the drug-specified, is a strategy to advance patient care more quickly and efficiently. The idea was promoted in 2012 through the Discovering New Therapeutic Uses for Existing Molecules program, initiated by NIH. Several drugs used for other purposes have proved to be effective anti-cancer agents, including thalidomide, aspirin, and celecoxib. In contrast, novel drug development is a lengthy process. Molecularly tailored drugs fail for various reasons including inadequate bioavailability and adverse side effects, and are often very expensive. Despite drawbacks to targeted drug development, there are also successes in this area of research.

Cancer therapeutics designed to inhibit kinases involved in cell growth and survival pathways are potent and selective in the short term. By directly blocking cell signaling, often at the source of over-activation, these drugs kill cancer cells. One example of success in targeted therapy is the development of BRAFV600E inhibitors, vemurafenib and dabrafenib, and their ability to prolong progression-free survival time in melanoma patients. However, a major problem with monotherapy is the inevitable development of resistance. Even with combination treatment using specific inhibitors, such as BRAFV600E and MEK inhibitors in melanoma, improvement in patient outcome is limited [1,2] and resistance emerges.

There is increasing interest in combining targeted inhibitors with common metabolic regulators to hinder resistance. This unique combination reaps both the intrinsic benefits of repurposing drugs and the selectivity of targeted therapy. The major biguanides, metformin and phenformin, have known pharmacokinetics, high safety profiles, and are relatively inexpensive. Metformin in particular is widely used for treatment of patients with type 2 diabetes. Although there is evidence in literature for both pro-cancer and anti-cancer effects of metformin on cancer cells, a clear association between metformin therapy and reduced risk of cancer in diabetic patients exists. Recent studies point out direct cellular benefits of combining biguanides with current targeted therapy.

Biguanides in combination with targeted inhibitors synergistically reduced cell viability and inhibited tumor growth in *BRAFV600E*-positive and *NRAS* mutant melanoma cells [3,4]. In the latter case, even when the driver *NRAS* mutation was not targeted itself, inhibition of a downstream molecule was more effective when used in combination with metformin [4]. Another study determined that metformin and erlotinib (EGFR inhibitor) induced apoptosis synergistically in a subset of basal-like breast cancer cells. Specifically, MDA-MB-468 cells treated with the combination had reduced expression of signaling molecules, increased apoptosis, and reduced clonogenicity and mammosphere formation [5]. Our current finding of the increased sensitivity of thyroid cancer cell lines to the cytotoxic activity of vemurafenib, when used in combination with metformin [6], provides further evidence of such synergism that can potentially be useful in cancer treatment.

Molecular mechanisms accounting for biguanide-mediated potentiation of targeted inhibitors likely include pleiotropic effects from altering cancer cell metabolism and inhibition of the mammalian target of rapamycin (mTOR) molecule. Metformin transiently inhibits the mitochondrial respiratory-chain complex 1, causing decreased energy status, increased ratio of AMP:ATP, and subsequent activation of AMP-activated protein kinase (AMPK), which has many downstream targets. Overall, the cell's agenda changes to an energy-sparing, relatively anabolic state. There is evidence for metformin-mediated decreased fatty acid synthesis, inhibition of the unfolded protein response, and cell cycle arrest. Broad metabolic modifications may help to restrict development of resistance: less selective pressure deters bypass of a single inhibited molecule and multiple adaptations may be required for resistant cell survival. Our observation that rapamycin, like metformin, also enhances cancer cell sensitivity to vemurafenib [6] emphasizes the importance of mTOR signaling and expands the range of potential adjuvants to include direct and specific mTOR inhibitors.

Inhibition of mTOR signaling reduces translation and inhibits cell growth and proliferation. In cancer cells, these actions oppose overactive growth-promoting signaling pathways that are often inhibited by targeted therapy. Inhibition of mTOR creates a further downstream block of growth signaling and may prevent resistance from developing through alternative upstream pathways. The idea of a convergence point of resistance was elucidated in a recent study, which found that resistance to targeted inhibitors was characterized by persistent formation of the eIF4F translation initiation complex in melanoma, colon, and thyroid cancer cells [7]. Furthermore, disruption of eIF4F complex formation in combination with targeted inhibitors caused synergistic cell death [7]. Since metformin decreases translation initiation through inhibition of mTOR, it could also evoke synergy through inhibition at the convergence point.

Biguanide adjuvant therapy is a rationale and cost-effective strategy to improve upon outcomes in cancer patients treated with targeted therapies. Further investigation into effectiveness of this combination *in vivo* may lead to widespread treatment opportunities in the near future.
